# Association between serum calcium levels and first stroke: A community-based nested case-control study

**DOI:** 10.3389/fneur.2022.938794

**Published:** 2022-08-05

**Authors:** Congcong Ding, Chonglei Bi, Tengfei Lin, Lishun Liu, Yun Song, Binyan Wang, Ping Wang, Chongqian Fang, Hai Ma, Xiao Huang, Xiping Xu, Hao Zhang, Lihua Hu, Yong Huo, Xiaobin Wang, Huihui Bao, Xiaoshu Cheng

**Affiliations:** ^1^Department of Cardiovascular Medicine, The Second Affiliated Hospital of Nanchang University, Nanchang, China; ^2^Center for Prevention and Treatment of Cardiovascular Diseases, The Second Affiliated Hospital of Nanchang University, Nanchang, China; ^3^People's Hospital of Rongcheng, Rongcheng, China; ^4^Beijing Advanced Innovation Center for Food Nutrition and Human Health, College of Food Science and Nutritional Engineering, China Agricultural University, Beijing, China; ^5^Institute of Biomedicine, Anhui Medical University, Hefei, China; ^6^Department of Research, Shenzhen Evergreen Medical Institute, Shenzhen, China; ^7^School of Public Health (Shenzhen), Sun Yat-sen University, Guangzhou, China; ^8^Health and Family Planning Commission, Rongcheng, China; ^9^Department of Cardiology, Peking University First Hospital, Beijing, China; ^10^Department of Population, Family and Reproductive Health, Johns Hopkins University Bloomberg School of Public Health, Baltimore, MD, United States

**Keywords:** calcium, first stroke, ischemic stroke, U-shaped curve, Chinese population

## Abstract

**Background:**

Evidence from epidemiologic studies has been limited and inconsistent regarding the role of serum calcium in stroke incidence risk. We aimed to evaluate the association between serum albumin-corrected calcium and the risk of the first stroke in the Chinese community-dwelling population.

**Methods:**

The study sample population was drawn from the “H-type Hypertension and Stroke Prevention and Control Project.” Using a nested case-control study, a total of 1,255 first-stroke cases and 1,255 controls matched for age, sex, and village were included in the final data analysis. We measured the serum calcium by inductively coupled plasma mass spectrometry and assessed the associations between serum albumin-corrected calcium and first stroke using conditional logistic regression.

**Results:**

The overall mean (SD) serum albumin-corrected calcium was 8.9 (0.6) mg/dl. Compared with the middle tertile (8.7–9.1 mg/dl), the multivariate-adjusted odds ratios (95% CIs) of first total stroke associated with the lowest tertile and the highest tertile of serum albumin-corrected calcium were 1.37 (1.10, 1.70) and 1.30 (1.04, 1.62), respectively. Similar trends were observed for the first ischemic stroke. Consistently, restricted cubic spline showed a U-shaped association between serum albumin-corrected calcium and risk of total stroke and ischemic stroke. However, serum albumin-corrected calcium had no significant effect on first hemorrhagic stroke. No significant effect modification was observed in the subgroup analysis.

**Conclusions:**

Our results suggested a U-shaped association between serum calcium and first stroke; both low and high serum calcium levels were associated with an increased risk of the first stroke in the Chinese population.

## Introduction

Stroke is the leading cause of death in China and the second leading cause of death in the world (1, 2). There is an urgent need to identify novel modifiable risk factors to inform the primary prevention of stroke because about 77% of strokes are first events (3). Recently, there has been increasing interest in the effects of minerals and trace elements on the risk of cardiovascular diseases (CVDs) (4–6).

Calcium is the most abundant mineral in the body. Besides being the structural component of bone, calcium plays a very important role in the coagulation system, intracellular signaling and muscle contraction, and nervous system function (7). Nevertheless, it is also associated with certain pathological processes such as atherosclerotic plaques, and other soft-tissue calcifications (8, 9). Previous studies have reported that serum calcium levels were associated with cardiovascular risk factors such as hypertension, diabetes, and dyslipidemia (10, 11). Therefore, several functions and disease associations of calcium are postulated to affect the cardiovascular system. However, evidence from observational or genetic epidemiologic studies has been limited and conflicting. Some studies demonstrated an increased risk of stroke with elevated serum calcium (12, 13), while others show null associations for stroke (7), or inverse associations (14). Moreover, few previous studies have comprehensively analyzed the underlying modifiers of the association between calcium and stroke risk.

Our current study aimed to evaluate the relation of serum calcium with the risk of first total stroke and stroke subtypes (ischemic stroke and hemorrhagic strokes) and to examine any possible effect modifiers using data from a community-based population in China.

## Methods

### Study design and population

The current study was a substudy of the “H-type Hypertension and Stroke Prevention and Control Project (HSPCP),” which was an ongoing, multicenter, prospective, community-based, noninterventional, and observational, real-world registry study and was mainly conducted in Rongcheng, Shandong province, and Lianyungang, Jiangsu province, China (15). Aiming to investigate the prevalence and treatment of H-type hypertension in China, explore risk factors, and construct a risk prediction model of cardiovascular, cerebrovascular, and renal diseases, the HSPCP was conducted with the following criteria for selecting eligible participants: men and women with age more than 18 years and essential hypertension which defined as resting SBP (systolic blood pressure) ≥140 mmHg or seated DBP (diastolic blood pressure) ≥90 mmHg at screening. Subjects who were not able to complete informed consent or who were unable to be visited according to study protocol because of psychological or nervous system damage were excluded from the current study.

From January to December 2017, 115,337 patients with hypertension in Rongcheng city, Shandong province were screened and their baseline general information and blood samples were collected. Data were linked to stroke data through 31 December 2018. A nested case-control study design was performed in the current study with 1,401 stroke cases and 1,401 controls. We performed a 1:1 matching of cases to controls (nonstroke) for age in the range of ±1 years and exact match for sex and village. Controls were selected who were still alive at follow-up and who never had a stroke before the study and during the follow-up period and were matched with incident stroke cases. Propensity score matching analysis was not used in our study. Patients with stroke having complete medical records from the Chinese Centers for Disease Control and Prevention (CDC, 2016–2018) were selected as cases. After excluding 287 cases without baseline serum calcium value, and 5 unpaired individual data, 1,255 stroke cases, and 1,255 controls were entered into the final analysis (Supplementary Figure 1).

### Ethics

The current study was approved by the Ethics Committee of Lianyungang Precision Health Institute, the Ethics Committee of the Institute of Biomedicine, Anhui Medical University, Hefei, China, and the Ethics Committee of the Lianyungang Center for Advanced Research in Cardiovascular Diseases. Signed written consents were provided by all participants.

### Outcomes

The primary outcome of this study was new-onset total stroke (fatal and nonfatal) including first ischemic stroke (I63), first hemorrhagic stroke (I60-I61), and undetermined stroke (I64). Secondary outcomes included first ischemic stroke (fatal and nonfatal), and first hemorrhagic stroke (fatal and nonfatal). All diseases were coded according to ICD-10 (International Classification of Diseases, 10th Revision). Incidences of stroke were obtained from CDC, Rongcheng, China. Collected stroke data were further validated by checking the national health insurance system which was linked with all hospitalization records, and follow-up visits.

All information about new stroke cases, which included demographic information, diagnostic bases, and time, was required to report to the local center for disease control and prevention by government-issued documents. Quality control was accomplished by trained officials to check errors, determine missing cases, and delete repeated data, which was also a responsibility of the local center for disease control and prevention. Moreover, 5% of total stroke cases were randomly chosen for further confirmation through phone calls or in-door visits.

### Laboratory assays

The fasting blood sample of each participant was collected at baseline. Albumin, phosphate, total homocysteine, fasting lipid, and glucose concentrations at baseline were measured using automatic clinical analyzers (Beckman Coulter AU680) at the core laboratory. Levels of 25 hydroxyvitamin D3 [25(OH)D_3_] were measured by liquid chromatography with tandem quadrupole mass spectrometry (LC-MS/MS), while calcium and magnesium levels were measured by inductively coupled plasma mass spectrometry (ICP-MS) (16, 17) using Thermo Fisher iCAP Q ICP-MS in a commercial lab (Beijing DIAN Medical Diagnostics Laboratory, China). Both intra-assay and inter-assay CVs of duplicate samples (randomly placed among the study samples) were calculated. The intra-assay CV for calcium is ranged from 1.08 to 8.16%, while the inter-assay CV for calcium is ranged from 2.76 to 3.59%. As calcium is mostly bound to albumin in the blood, calcium (mg/dl) values were corrected for based on the serum albumin (g/dl) values according to the Payne formula: albumin-corrected calcium (mg/dl) = calcium + 0.8 × (4.0 − albumin) (18).

The estimated glomerular filtration rate (eGFR) was calculated with the use of the Chronic Kidney Disease Epidemiology Collaboration (CKD-EPI) equation (19).

### Statistical analysis

Data are presented as mean ± SD for continuous variables and as frequency (%) for categorical variables. Differences in baseline characteristics between cases and controls were compared using chi-square tests for categorical variables and *t*-tests for continuous variables. Variables that are known as traditional or suspected risk factors for stroke (20), matched variables, or variables that showed significant differences between cases and controls were chosen as the covariates in our current study. Odds ratios (ORs) of total stroke, ischemic stroke, and hemorrhagic stroke were estimated by modeling serum albumin-corrected calcium as tertiles using conditional logistic regression. Potential confounders included in the logistic regression models, other than the matching criteria (sex, age, and village), were body mass index (BMI), SBP, DBP, smoking status, drinking status, labor intensity, total cholesterol, triglycerides, high-density lipoprotein cholesterol (HDL-C), fasting glucose, total homocysteine, eGFR, phosphate, 25(OH)D_3_, magnesium, antihypertensive drugs, glucose-lowering drugs, lipid-lowering drugs, and antiplatelet drugs. To further characterize the shape of the relationship between serum albumin-corrected calcium and first stroke and its subtypes, we also used a generalized additive model (GAM) and smooth curve fitting (penalized spline method). In addition, possible modifications of the association between serum albumin-corrected calcium and first stroke were also assessed for the following variables: sex, age (<65 compared with ≥65 years), BMI (<24 compared with ≥ 24 kg/m^2^), current smoking (yes compared with no), current alcohol drinking (yes compared with no), fasting glucose (<7.0 compared with ≥7.0 mmol/L or history of diabetes), total cholesterol [<5.8 (median) compared with ≥5.8 mmol/L], SBP [<151.3 (median) compared with ≥151.3 mmHg], magnesium [< 19.9 (median) compared with ≥ 19.9 mg/L], phosphate [<1.2 (median) compared with ≥ 1.2 mmol/L], and 25(OH)D_3_ [< 23.2 (median) compared with ≥23.2 ng/ml]. Potential interactions were examined by including the interaction terms in those logistic regression models with the greatest number of confounding variables.

A two-tailed *P* < 0.05 was considered to be statistically significant in all analyses. R software version 3.4.3 (www.R-project.org) and Empower version 2.17.8 (www.empowerstats.com, X&Y Solutions, Inc.) were used for all statistical.

## Results

### Study participants and baseline characteristics

**Figure 1:**
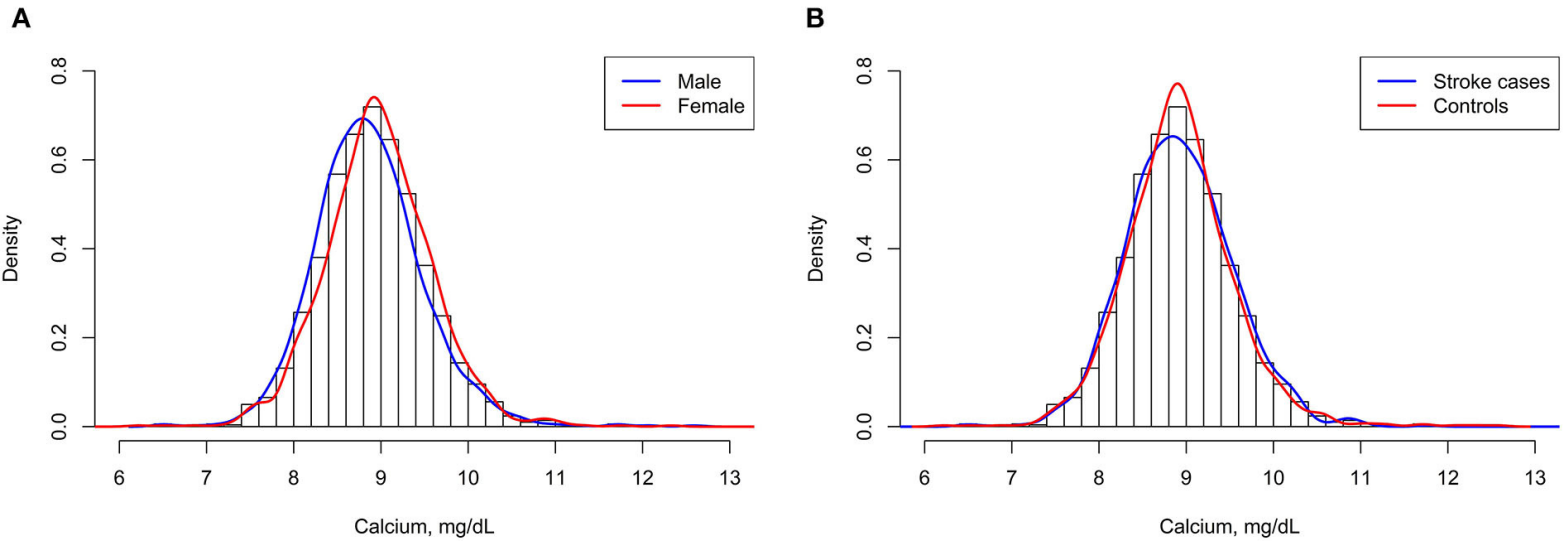
Serum albumin-corrected calcium distributions of men and women **(A)** and stroke cases and control subjects **(B)**.

A total of 1,255 stroke cases were diagnosed during the period from the time of the initial baseline blood collection until the end of follow-up, and 1,255 matched controls were analyzed finally in this study. Among the 1,255 incident stroke cases, 1,079 were ischemic strokes, 171 were hemorrhagic strokes, and 5 were undetermined strokes. Overall, the mean (SD) age was 70.8 (8.1) years and 49.5% were men. Mean ± SD values of serum albumin-corrected calcium was 8.9 ± 0.6 mg/dl (men: 8.9 ± 0.6 mg/dl; women: 9.0 ± 0.6 mg/dl, *P* < 0.05). Accordingly, as shown in Figure 1A, there was a clear shift toward the right in the distribution of serum albumin-corrected calcium in women compared with men. The distribution of serum albumin-corrected calcium in the case subjects was similar to that in the control subjects (Figure 1B).

Baseline characteristics of cases and control participants were shown in Table 1. Mean ± SD values of serum albumin-corrected calcium were 8.9 ± 0.6 mg/dl both in case and control subjects and there were no statistically significant differences (*P* > 0.05). In addition, stroke cases tended to have higher BMI, SBP, DBP, triglycerides, total homocysteine, and fasting glucose; have a higher rate of current smokers, antihypertensive drugs, glucose-lowering drugs, and antiplatelet drugs; and have lower labor intensity, eGFR, and HDL-C levels (all *P* < 0.05). No significant differences were found between the two groups in terms of age, sex, drinking status, albumin, total cholesterol, phosphate, 25(OH)D_3_, magnesium levels, and use of lipid-lowering drugs.

**Table 1:** Baseline characteristics of cases and control subjects^*^.

	**First stroke cases** **(*n =* 1,255)**	**Non-stroke** **controls** **(*n =* 1,255)**	***P-*value**
Age, y	70.8 ± 8.1	70.8 ± 8.1	0.987
Male, *n* (%)	621 (49.5%)	621 (49.5%)	1.000
BMI, kg/m^2^	26.5 ± 4.4	25.9 ± 3.7	<0.001
SBP, mmHg	157.2 ± 23.8	149.3 ± 21.7	<0.001
DBP, mmHg	87.2 ± 12.8	83.3 ± 11.4	<0.001
Current smoking, *n* (%)	296 (23.6%)	255 (20.3%)	0.048
Current alcohol drinking, *n* (%)	295 (23.5%)	317 (25.3%)	0.306
Labor intensity, *n* (%)			0.003
Mild	978 (77.9%)	907 (72.3%)	
Moderate	221 (17.6%)	267 (21.3%)	
Severe	56 (4.5%)	81 (6.5%)	
**Laboratory results**			
Albumin, g/dL	4.7 ± 0.3	4.7 ± 0.3	0.180
Total cholesterol, mmol/L	5.8 ± 1.2	5.9 ± 1.2	0.768
Triglycerides, mmol/L	1.5 ± 0.9	1.3 ± 0.8	<0.001
HDL-C, mmol/L	1.6 ± 0.4	1.7 ± 0.4	<0.001
Total homocysteine, μmol/L	14.3 ± 8.0	13.5 ± 6.2	0.006
Fasting glucose, mmol/L	6.5 ± 2.5	6.0 ± 2.1	<0.001
eGFR, mL min^−1^ 1.73 m^−2^	91.7 ± 15.3	93.3 ± 13.4	0.005
Phosphate, mmol/L	1.2 ± 0.3	1.2 ± 0.2	0.563
25(OH)D_3_, ng/mL	24.1 ± 9.1	24.4 ± 9.1	0.422
Magnesium, mg/L	19.9 ± 1.9	20.0 ± 1.8	0.123
Calcium, mg/dL	9.5 ± 0.6	9.5 ± 0.6	0.550
Albumin-corrected calcium, mg/dL	8.9 ± 0.6	8.9 ± 0.6	0.955
**Medication use**, ***n*** **(%)**			
Antihypertensive drugs	664 (52.9%)	488 (38.9%)	<0.001
Glucose-lowering drugs	193 (15.4%)	108 (8.6%)	<0.001
Lipid-lowering drugs	22 (1.8%)	22 (1.8%)	1.000
Antiplatelet drugs	60 (4.8%)	23 (1.8%)	<0.001

* For continuous variables, values are presented as means (SD). BMI, body mass index; SBP, systolic blood pressure; DBP, diastolic blood pressure; HDL-C, high-density lipoprotein cholesterol; eGFR, estimated glomerular filtration rate; 25 hydroxyvitamin D3, 25(OH)D_3_.

Furthermore, participants with higher serum albumin-corrected calcium levels tended to be women, had higher age, triglycerides, phosphate, 25(OH)D_3_, magnesium levels, lower albumin, eGFR levels, and were less likely to consume alcohol (all *P* < 0.05) (Supplementary Table 1).

### Association between calcium and first stroke

The outcome was first stroke (average time of first stroke, 0.8 years). Table 2 shows the association of first stroke and its subtypes with serum corrected calcium using conditional logistic regression analyses. When serum corrected calcium was assessed as tertiles, compared with the middle tertile (8.7–9.1 mg/dl), the multivariate-adjusted ORs (95% CIs) of first total stroke significantly associated with the lowest tertile and the highest tertile of serum albumin-corrected calcium were 1.37 (1.10, 1.70) and 1.30 (1.04, 1.62), respectively. Similar trends were observed for first ischemic stroke; both low and high levels of serum albumin-corrected calcium were significantly associated with higher odds of first ischemic stroke. The corresponding ORs (95% CIs) for the first ischemic stroke were 1.41 (1.11, 1.79) and 1.34 (1.05, 1.71), respectively. However, there was no significant association between either low or high tertile of serum-corrected calcium and the risk of first hemorrhagic stroke (OR: 1.22, 95% CI: 0.65, 2.30; OR: 0.87, 95% CI: 0.46, 1.63, respectively).

**Table 2:** Risk of first stroke (total and subtypes) associated with serum albumin-corrected calcium levels^*^.

**Albumin-corrected** **calcium, mg/dL**	**Cases/controls**	**Model 1**		**Model 2**	
		**OR (95% CI)**	***P***	**OR (95% CI)**	***P***
**First stroke**					
**Tertiles**					
T1 (<8.7)	440/397	1.35 (1.11, 1.65)	0.003	1.37 (1.10, 1.70)	0.005
T2 (8.7–9.1)	379/457	Ref.		Ref.	
T3 (≥9.1)	436/401	1.33 (1.09, 1.62)	0.005	1.30 (1.04, 1.62)	0.020
**Ischemic stroke**					
**Tertiles**					
T1 (<8.7)	376/342	1.35 (1.09, 1.67)	0.006	1.41 (1.11, 1.79)	0.005
T2 (8.7–9.1)	327/396	Ref.		Ref.	
T3 (≥9.1)	376/341	1.36 (1.10, 1.69)	0.005	1.34 (1.05, 1.71)	0.017
**Hemorrhagic stroke**					
**Tertiles**					
T1 (<8.7)	62/53	1.43 (0.82, 2.48)	0.210	1.22 (0.65, 2.30)	0.535
T2 (8.7–9.1)	51/60	Ref.		Ref.	
T3 (≥9.1)	58/58	1.17 (0.70, 1.95)	0.561	0.87 (0.46, 1.63)	0.661

* Model 1 is conditioned on the matching factors of age, sex, and village.

Model 2 is conditioned on the matching factors of age, sex and village, and adjusted for BMI, SBP, DBP, smoking status, drinking status, labor intensity, total cholesterol, triglycerides, HDL-C, fasting glucose, total homocysteine, eGFR, phosphate, 25(OH)D3, magnesium, antihypertensive drugs, glucose-lowering drugs, lipid-lowering drugs, antiplatelet drugs.

**Figure 2:**
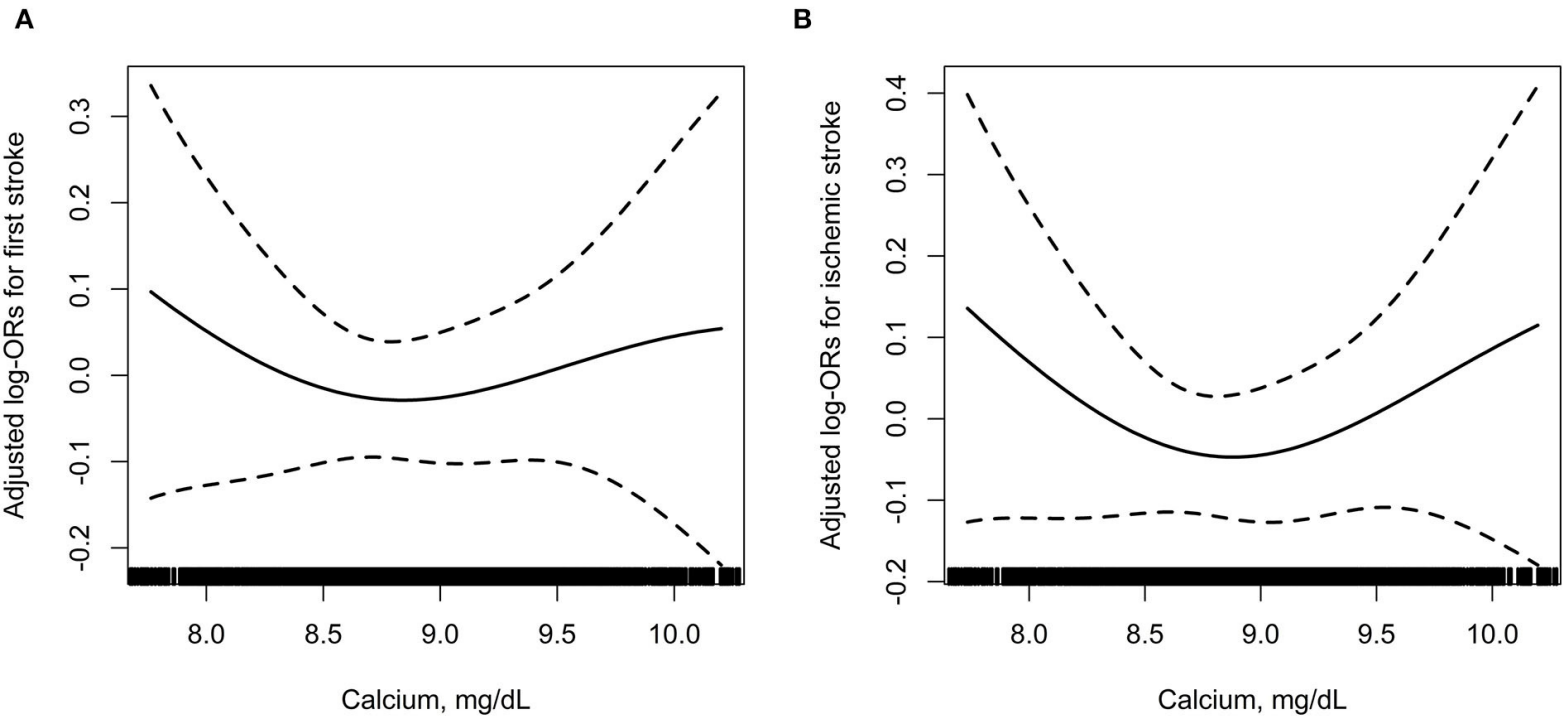
The association between baseline serum albumin-corrected calcium and incident risk of first total stroke **(A)** and ischemic stroke **(B)**. In addition to the matching factors (age, sex, and study village), the splines also adjusted for BMI, SBP, DBP, smoking status, drinking status, labor intensity, total cholesterol, triglycerides, HDL-C, fasting glucose, total homocysteine, eGFR, phosphate, 25(OH)D_3_, magnesium, antihypertensive drugs, glucose-lowering drugs, lipid-lowering drugs, and antiplatelet drugs.

Further analyses using restricted cubic spline confirmed the U-shaped association between serum albumin-corrected calcium and risk of first total stroke and ischemic stroke (Figure 2).

### Subgroup analyses

We further explored the role of other covariables on the association between serum albumin-corrected calcium and first stroke. As shown in Table 3, none of the stratified variables, namely, sex (male vs. ≥ female; *P*-interactio*n* = 0.790), age (<65 vs. ≥ 65 years; *P*-interactio*n* = 0.690), BMI (<24 vs. ≥ 24 kg/m^2^; *P*-interactio*n* = 0.607), current smoking (yes vs. no; *P*-interactio*n* = 0.824), current alcohol drinking (yes vs. no; *P*-interactio*n* = 0.681), fasting glucose (<7.0 vs. ≥7.0 mmol/L or history of diabetes; *P*-interactio*n* = 0.889), total cholesterol (<5.8 vs. ≥5.8 mmol/L; *P*-interactio*n* = 0.615), SBP (<151.3 vs. ≥151.3 mmHg; *P*-interactio*n* = 0.846), magnesium (<19.9 vs. ≥19.9 mg/L; *P*-interactio*n* = 0.808), phosphate (<1.2 vs. ≥1.2 mmol/L; *P*-interactio*n* = 0.993), and 25(OH)D_3_ (<23.2 vs. ≥23.2 ng/ml; *P*-interactio*n* = 0.526), significantly modified the association between serum albumin-corrected calcium and the risk of first stroke (all *P* for interaction >0.05).

**Table 3:** The associations between serum albumin-corrected calcium and the risk of first stroke in various subgroups^*^.

**Subgroups**	**Cases/controls**	**OR (95% CI) of calcium tertiles**			***P* for interaction**
		**T1 (<8.7)**	**T2 (8.7–9.1)**	**T3 (≥9.1)**	
Sex					0.790
Male	621/621	1.39 (1.05, 1.85)	Ref.	1.39 (1.03, 1.89)	
Female	634/634	1.36 (1.01, 1.83)	Ref.	1.24 (0.94, 1.64)	
Age, y					0.690
<65	304/304	1.34 (0.88, 2.04)	Ref.	1.14 (0.73, 1.78)	
≥65	951/951	1.38 (1.09, 1.75)	Ref.	1.37 (1.09, 1.72)	
BMI, kg/m2					0.607
<24	328/404	1.12 (0.76, 1.65)	Ref.	1.17 (0.81, 1.70)	
≥24	927/851	1.47 (1.15, 1.87)	Ref.	1.38 (1.08, 1.76)	
Current smoking					0.824
No	959/1000	1.37 (1.09, 1.73)	Ref.	1.34 (1.07, 1.69)	
Yes	296/255	1.30 (0.83, 2.04)	Ref.	1.09 (0.68, 1.73)	
Current alcohol drinking					0.681
No	960/938	1.30 (1.02, 1.65)	Ref.	1.28 (1.02, 1.62)	
Yes	295/317	1.58 (1.04, 2.38)	Ref.	1.45 (0.93, 2.26)	
Fasting glucose, mmol/L					0.889
<7.0	896/1032	1.35 (1.07, 1.70)	Ref.	1.26 (1.00, 1.59)	
≥7.0 or history of diabetes^#^	359/223	1.37 (0.88, 2.12)	Ref.	1.43 (0.92, 2.23)	
Total cholesterol, mmol/L					0.615
<5.8 (median)	641/613	1.27 (0.96, 1.69)	Ref.	1.35 (1.01, 1.80)	
≥5.8	614/642	1.49 (1.10, 2.01)	Ref.	1.28 (0.96, 1.72)	
SBP, mmHg					0.846
<151.3 (median)	528/721	1.39 (1.04, 1.86)	Ref.	1.38 (1.03, 1.85)	
≥151.3	727/534	1.34 (1.00, 1.80)	Ref.	1.22 (0.91, 1.62)	
Magnesium, mg/L					0.808
<19.9 (median)	654/601	1.30 (0.99, 1.71)	Ref.	1.29 (0.94, 1.76)	
≥19.9	601/654	1.39 (1.02, 1.90)	Ref.	1.30 (0.99, 1.71)	
Phosphate, mmol/L					0.993
<1.2 (median)	590/593	1.33 (0.99, 1.78)	Ref.	1.28 (0.94, 1.73)	
≥1.2	665/662	1.41 (1.05, 1.89)	Ref.	1.33 (1.01, 1.76)	
25(OH)D_3_, ng/mL					0.526
<23.2 (median)	635/616	1.35 (1.01, 1.80)	Ref.	1.21 (0.90, 1.62)	
≥23.2	620/639	1.34 (1.00, 1.80)	Ref.	1.41 (1.06, 1.89)	

* ORs of first stroke in relation to serum albumin-corrected calcium levels (tertiles) were calculated using multivariate logistic regression models. Each subgroup analysis adjusted, if not stratified, for age, sex, BMI, BMI, SBP, DBP, smoking status, drinking status, labor intensity, total cholesterol, triglycerides, HDL-C, fasting glucose, total homocysteine, eGFR, phosphate, 25(OH)D3, magnesium, antihypertensive drugs, glucose-lowering drugs, lipid-lowering drugs, antiplatelet drugs, except for the stratifying variable.

# History of diabetes was defined as self-reported diabetes, or use of anti-diabetic medications.

## Discussion

In this nested case-control study, we first demonstrated a U-shaped relationship between serum calcium levels with risk of first stroke and ischemic stroke; both low and high serum calcium levels were associated with an increased risk of the first stroke in the Chinese community population. In addition, the U-shaped association was robust among various subgroups.

To date, research findings on the association between serum calcium and stroke risks remain inconclusive. A prospective community-based study, based on 552 stroke cases with 12.6 years of follow-up, found that serum calcium was significantly positively associated with stroke (per SD increment—hazard ratio [HR]: 1.16; 95% CI: 1.07, 1.26) in adults with higher average levels of corrected calcium (10.3 mg/dl) at baseline (12). Another *post hoc* data analysis from randomized controlled trial, conducted in 7,259 postmenopausal women with osteoporosis, reported that higher baseline serum calcium levels was associated with upward trends in stroke events (per SD increment—HR: 1.22; 95% CI: 0.99, 1.49; *P* = 0.06) (21). Rohrmann et al. (13) reported that serum-corrected calcium was positively associated with incident ischemic stroke, but not with incident hemorrhagic stroke when considering subtypes of total stroke in the Swedish AMORIS cohort. However, several reports have also yielded some conflicting results. Marniemi et al. (22) performed a case-control study on elderly subjects and demonstrated that high serum calcium had no significant effect on the risk of stroke [highest compared with the lowest tertile—risk ratio (RR): 1.15; 95% CI: 0.62, 2.12]. Lu et al. (23) also found that serum-corrected calcium was not associated with incident ischemic stroke in a large cohort of US veterans. Moreover, a recent genetic epidemiologic study reported that there was no association of genetically predicted serum calcium levels with all ischemic stroke [per 0.5 mg/dl (about 1 SD) increase in serum calcium: OR 1.03, 95%CI 0.88–1.21] or with any ischemic stroke subtype, using data from the MEGASTROKE consortium (34,217 ischemic stroke cases and 404,630 noncases) (7). In contrast, one study reported that an inverse association of serum calcium levels with ischemic stroke was observed (highest compared with the lowest tertile—HR, 0.73; 95% CI, 0.53–0.99; *P*_linear−trend_ = 0.013) in the case-cohort analysis (14).

These conflicting results might be attributed to the differences in observational study characteristics, sample size, adjustment of confounders, stroke subtype, serum calcium levels, and their measurement method and whether calcium was corrected or not. In this study, the ICP-MS method, a new elemental analysis technique, may be used as a candidate reference method for the standardization of serum calcium determination (16). Compared with the traditional colorimetric method (12, 13, 21) or enzymatic method (14) used in the above contradictory studies, the ICP-MS method not only can simultaneously measure multiple elements but also has higher sensitivity and wider limits of detection (24). This explained to some extent our inconsistency with previous results. Besides, in contrast to some other studies, we were able to take into account albumin concentration. Since a large part of the measured calcium is bound to albumin and is therefore not bioactive, serum albumin-corrected calcium levels are a better estimate of the bioactive calcium component (18).

In addition, although previous studies have been inconsistent, several related studies support our observations. Some studies showed that both reduced and increased serum-corrected calcium levels (including within the “normal” range) are associated with a higher risk of in-hospital and long-term mortality in patients with acute myocardial infarction (25, 26). What's more, in a large meta-analysis of 11 prospective studies, including a total of 757,304 individuals, a U-shaped relationship between dietary calcium intake and CVD mortality was also observed. Both low and high intakes were associated with an increased risk of CVD mortality compared with 800 mg/day (27). Similar to ours, these prior studies suggested that both higher and lower levels of serum calcium and calcium intake are associated with adverse outcomes.

The mechanisms of the U-shaped association between serum calcium and stroke could be explained by the complex and variable effect mechanism of calcium driven by alterations in concentration. First, low calcium levels could increase the calcium channels on vascular smooth muscle cells and allow calcium to flow into cells, leading to intracellular calcium overload (28). Intracellular calcium overload in platelet is one of the most important links in the process of atherosclerotic plaque formation or thrombogenesis (29). On the other hand, calcium influx is associated with dysfunction of vascular endothelial cells, increased lipid disposition, and inflammation (28). Second, calcium plays a vital role in the electrophysiologic stability of excitable cell membranes (23), and low serum calcium levels are associated with an increased risk of atrial fibrillation (30), which is one of the most common cardiac arrhythmia and a major cause of ischemic stroke. Third, low calcium levels are a sign of low vitamin D levels, since vitamin D could increase intestinal calcium absorption to maintain calcium homeostasis (31). Lower vitamin D has been consistently associated with incident stroke (32). In animal models, vitamin D may possess neuroprotective and antithrombotic properties and reduce ischemic cortical damage (33, 34). Consistently, our study showed participants with lower serum calcium who have lower 25(OH)D_3_ levels.

Conversely, higher serum calcium levels might increase the risk of CVD by promoting vascular calcification (9) and atherosclerosis (35). It was reported that higher serum calcium even within the normal range was associated with the presence and extent of intracranial atherosclerosis (35) which is considered a risk factor for ischemic stroke (36). Vascular calcification is a major prevalent pathology in atherosclerosis (37). Previous *in vitro* studies have shown that higher calcium could promote calcification of vascular smooth muscle cells by binding to calcium-sensing receptors (38), leading to the downregulation of calcium-sensing receptors and enhancing mineralization (39). Consistent with this, serum calcium is related to calcified rather than an uncalcified plaque in an observational study using coronary computed tomography angiography to evaluate plaque (40). Besides, cardiovascular risk factors, namely, old age, dyslipidemia, hypertension, and diabetes may accelerate the process of vascular calcification, atherosclerosis, and plaque rupture in the setting of high calcium levels (40, 41). In the current study, it is worth noting that the elderly hypertensive population accounted for the majority. Another possible mechanism is that calcium may affect CVD risk by affecting the coagulation pathway. Calcium is a cofactor in the coagulation pathway and can regulate platelet function through calcium-sensing receptors (42). *In vitro*, ionized calcium is negatively related to the delay until clot initiation and positively related to clot strength (43). Bristow et al. (44) reported that calcium supplements may acutely increase blood coagulability in post-menopausal women. Calcium supplements are known to raise serum calcium levels (45). This also explained to some extent why some studies showed calcium supplements increase CVD risk, including stroke (46). However, these potential mechanisms need to be further studied in the future.

Several potential limitations of our study should be noted. First, we measured serum calcium levels only at baseline, without taking into account their possible variability over time. Second, although we controlled for multiple factors of calcium metabolism including magnesium, phosphate, and 25(OH)D_3_ in plasma, we could not rule out residual confounding such as parathyroid hormone concentrations. We also lacked information on dietary or supplementary calcium intake at baseline and during the course of follow-up. Third, we measured total calcium rather than ionized calcium which could better reflect the biologically active fraction in circulation. However, direct ionized calcium measurement is unwieldy and is not well performed in routine clinical practice. Fourth, our study was underpowered to investigate the impact of calcium on the risk of first hemorrhagic stroke. Finally, our study included the middle-aged and elderly Chinese population and the results may not be generalizable to young or other races.

## Conclusion

In conclusion, this nested case-control study derived from the Chinese community-based population showed a U-shaped association between serum calcium and first stroke; both low and high serum calcium levels were associated with an increased risk of first stroke and first ischemic stroke. Our results, if further confirmed, may provide useful data for clinical and nutritional guidelines on the primary prevention of the first stroke among the community-based population in China. Serum calcium may serve as a potentially modifiable risk factor and a possible biomarker for monitoring and intervention, and maintaining relatively normal calcium levels may contribute to the primary prevention of first stroke and ischemic stroke. Further longitudinal investigations are needed to confirm our findings and elucidate their mechanisms.

## Data availability statement

The raw data supporting the conclusions of this article will be made available by the authors, upon reasonable request.

## Ethics statement

The study was approved by the Ethics Committee of Lianyungang Precision Health Institute, the Ethics Committee of the Institute of Biomedicine, Anhui Medical University, Hefei, China, and the Ethics Committee of the Lianyungang Center for Advanced Research in Cardiovascular Diseases. The patients/participants provided their written informed consent to participate in this study.

## Author contributions

CD participated in the literature search, data analysis, data interpretation, and wrote the manuscript. CD, CB, TL, LL, YS, BW, PW, CF, HM, XH, XX, HZ, LH, and XW collected data. YH, XX, HB, and XC conceived the study and participated in its design and coordination. HB and XC participated in the study design and provided critical revision. All authors approved the final version.

## Funding

This study was supported by funding from the following: The National Key Research and Development Program [2016YFE0205400, 2018ZX09739010, and 2018ZX09301034003], the Science and Technology Planning Project of Guangzhou, China [201707020010], the Science, Technology and Innovation Committee of Shenzhen [GJHS20170314114526143 and JSGG20180703155802047], the Economic, Trade and Information Commission of Shenzhen Municipality [20170505161556110 and 20170505160926390], the National Natural Science Foundation of China [81960074 and 81500233], Jiangxi Outstanding Person Foundation [20192BCBL23024], and the major projects of the Science and Technology Department, Jiangxi [20171BAB205008].

## Conflict of interest

The authors declare that the research was conducted in the absence of any commercial or financial relationships that could be construed as a potential conflict of interest.

## Publisher's note

All claims expressed in this article are solely those of the authors and do not necessarily represent those of their affiliated organizations, or those of the publisher, the editors and the reviewers. Any product that may be evaluated in this article, or claim that may be made by its manufacturer, is not guaranteed or endorsed by the publisher.
