# Supplementary material for: Association between serum calcium levels and first stroke: A community-based nested case-control study
Source: Front Neurol. 2022 Aug 5;13:938794. doi: 10.3389/fneur.2022.938794 (PMC9388910; doi:10.3389/fneur.2022.938794)
Supplement: Supplementary file 1 [file Data_Sheet_1.doc]

**Supplementary Table 1. Baseline characteristics of the study participants by tertiles of serum albumin-corrected calcium***

| Characteristics | Serum albumin-corrected calcium, mg/dL | | | *P* value |
| --- | --- | --- | --- | --- |
|  | T1 (<8.7) | T2 (8.7 to 9.1) | T3 (≥9.1) |  |
| N | 837 | 836 | 837 |  |
| Age, y | 70.3 ± 7.7 | 70.4 ± 8.3 | 71.6 ± 8.1 | <0.001 |
| Male, n (%) | 470 (56.2%) | 408 (48.8%) | 364 (43.5%) | <0.001 |
| BMI, kg/m^2^ | 26.3 ± 4.0 | 26.0 ± 3.8 | 26.2 ± 4.5 | 0.390 |
| SBP, mmHg | 153.4 ± 22.9 | 152.4 ± 23.0 | 153.9 ± 23.4 | 0.370 |
| DBP, mmHg | 86.0 ± 12.4 | 84.9 ± 12.2 | 84.8 ± 12.2 | 0.070 |
| Current smoking, n (%) | 193 (23.1%) | 192 (23.0%) | 166 (19.8%) | 0.193 |
| Current alcohol drinking, n (%) | 239 (28.6%) | 190 (22.7%) | 183 (21.9%) | 0.002 |
| Labor intensity, n (%) |  |  |  | 0.449 |
| Mild | 617 (73.7%) | 646 (77.3%) | 622 (74.3%) |  |
| Moderate | 171 (20.4%) | 146 (17.5%) | 171 (20.4%) |  |
| Severe | 49 (5.9%) | 44 (5.3%) | 44 (5.3%) |  |
| Laboratory results |  |  |  |  |
| Albumin, g/dL | 4.8 ± 0.3 | 4.7 ± 0.3 | 4.6 ± 0.4 | <0.001 |
| Total cholesterol, mmol/L | 5.8 ± 1.2 | 5.8 ± 1.2 | 5.9 ± 1.2 | 0.086 |
| Triglycerides, mmol/L | 1.3 ± 0.8 | 1.4 ± 0.8 | 1.5 ± 0.9 | <0.001 |
| HDL-C, mmol/L | 1.6 ± 0.4 | 1.6 ± 0.4 | 1.6 ± 0.4 | 0.455 |
| Total homocysteine, μmol/L | 14.2 ± 8.6 | 13.4 ± 5.2 | 14.0 ± 7.2 | 0.058 |
| Fasting glucose, mmol/L | 6.3 ± 2.3 | 6.2 ± 2.2 | 6.2 ± 2.5 | 0.667 |
| eGFR, mL · min^−1^ · 1.73 m^−2^ | 93.8 ± 13.4 | 92.7 ± 14.8 | 91.0 ± 14.8 | <0.001 |
| Phosphate, mmol/L | 1.2 ± 0.3 | 1.2 ± 0.2 | 1.2 ± 0.3 | 0.047 |
| 25(OH)D_3_, ng/mL | 23.7 ± 8.7 | 24.0 ± 9.0 | 24.9 ± 9.6 | 0.033 |
| Magnesium, mg/L | 19.3 ± 1.7 | 20.0 ± 1.8 | 20.4 ± 2.0 | <0.001 |
| Medication use, n (%) |  |  |  |  |
| Antihypertensive drugs | 379 (45.3%) | 385 (46.1%) | 388 (46.4%) | 0.902 |
| Glucose-lowering drugs | 102 (12.2%) | 97 (11.6%) | 102 (12.2%) | 0.914 |
| Lipid-lowering drugs | 19 (2.3%) | 14 (1.7%) | 11 (1.3%) | 0.322 |
| Antiplatelet drugs | 27 (3.2%) | 28 (3.3%) | 28 (3.3%) | 0.987 |

*For continuous variables, values are presented as means (SD). Abbreviations: BMI, body mass index; SBP, systolic blood pressure; DBP, diastolic blood pressure; HDL-C, high-density lipoprotein cholesterol; eGFR, estimated glomerular filtration rate; 25 hydroxyvitamin D3, 25(OH)D_3_.

**
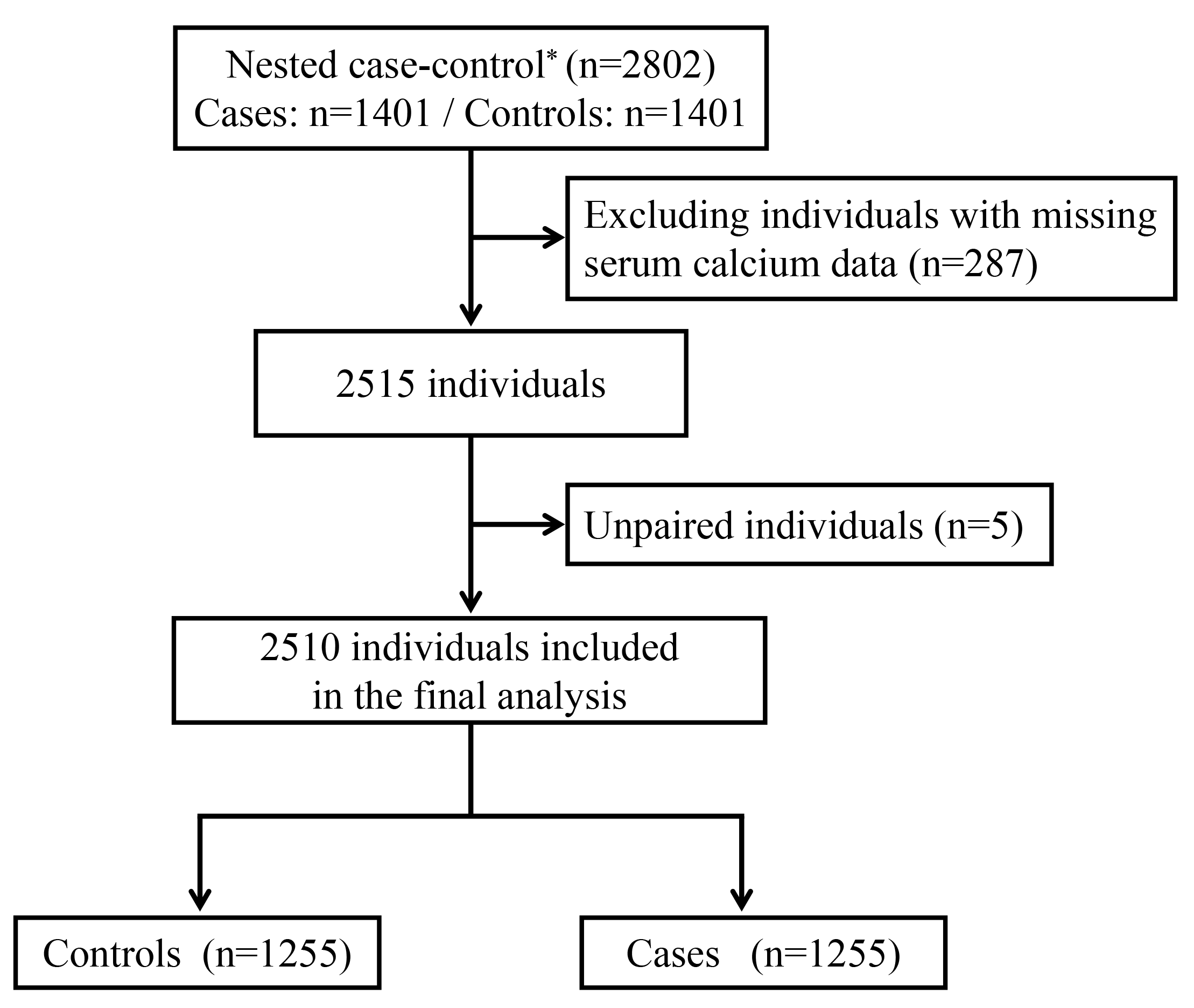
**

**Supplementary Figure 1. Flow chart of the study participants using a nested case-control design.** 1401 controls were individually matched with 1401 cases by age (within 1 year), sex and village on a 1:1 ratio.
